# A novel synthesized prodrug of gemcitabine based on oxygen-free radical sensitivity inhibited the growth of lung cancer cells

**DOI:** 10.7555/JBR.37.20230022

**Published:** 2023-09-28

**Authors:** Xinlu Chai, Yuting Meng, Wei Ge, Juan Wang, Fei Li, Xue Jun Wang, Xuerong Wang

**Affiliations:** 1 Department of Pharmacology, Nanjing Medical University, Nanjing, Jiangsu 210029, China; 2 Department of Pharmacology, Wannan Medical College, Wuhu, Anhui 241002, China; 3 Department of Pharmacy, Nanjing Medical University, Nanjing, Jiangsu 210029, China; 4 Department of Biochemistry and Molecular Biology, Nanjing Medical University, Nanjing, Jiangsu 210029, China

**Keywords:** gemcitabine, thiazolidinone, H _2_O _2_-sensitive moiety, non-small cell lung cancer

## Abstract

In the present study, we introduced the H _2_O _2_-sensitive thiazolidinone moiety at the 4th amino group of gemcitabine (GEM) to synthesize a new target compound named GEM-ZZQ, and then we confirmed its chemical structure by nuclear magnetic resonance spectroscopy. We further confirmed that GEM-ZZQ had a good chemical stability in different pH solutions *in vitro* and that it could be activated by H _2_O _2_ to release GEM. Pharmacodynamic studies revealed that the growth inhibition of human normal epithelial cells was weaker by GEM-ZZQ than by GEM treatment and that the inhibition of various lung cancer cell lines by GEM-ZZQ was similar to that of GEM. For the lung cancer cell lines that are resistant to the epidermal growth factor receptor (EGFR)-targeting inhibitor osimertinib, GEM-ZZQ showed less growth inhibition than GEM; however, GEM-ZZQ in combination with cisplatin showed better synergistic effects than GEM in the low-dose groups. In summary, we provided a new anti-cancer compound GEM-ZZQ for treating lung cancer by modifying the GEM structure.

## Introduction

Gemcitabine (GEM; 2′,2′-difluoro-2′-deoxycytidine, dFdC), a pyrimidine nucleoside analog ^[ 1] ^, was first synthesized by Eckel *et al* in 1988 and approved by FDA for pancreatic cancer treatment in 1996. Currently, GEM is also used for the treatment of non-small cell lung cancer (NSCLC) ^[ 2– 3] ^. According to the 2022 Chinese Society of Clinical Oncology (CSCO) and National Comprehensive Cancer Network (NCCN) guidelines for the treatment of NSCLC, GEM is the first-line treatment for locally advanced (stage Ⅲ) and metastatic (stage Ⅳ) NSCLC; and for patients who are resistant to various targeted therapeutic agents, GEM is still one of the first-line chemotherapy regimens.

GEM acts in cells mainly through its structural similarity to nucleoside deoxycytidine, and it has several action modes inside the cell. It is an antimetabolite and must be metabolized to the active phosphorylated form, gemcitabine triphosphate (dFdCTP) and gemcitabine diphosphate (dFdCDP), after being uptaken by cells. GEM is firstly phosphorylated by deoxycytidine kinase (dCK), which is a rate-limiting enzyme for dFdCDP and dFdCTP production, to form gemcitabine monophosphate (dFdCMP), and then dFdCMP is successively phosphorylated again by pyrimidine nucleoside monophosphate kinase or some other unknown enzymes to give dFdCDP. At last, dFdCDP may be catalyzed by nucleoside diphosphate kinase to become the final phosphorylated form dFdCTP. The most important mechanism of GEM action is the inhibition of DNA replication. Because the structure of dFdCTP is similar to dCTP, it can replace dCTP to be incorporated into the end of elongating DNA strands during DNA replication. Afterward, one more dNTP is added and then the DNA polymerases are unable to proceed anymore. Moreover, the proofreading enzymes are unable to remove this active metabolite of GEM from this position and the DNA strand elongation is stopped. This action (so-called masked termination) halts the DNA strand synthesis, and consequently induces cell apoptosis, and as well as exerts anti-tumor effects. In addition, dFdCDP can covalently bind to the active site of ribonucleotide reductase and inhibit its activity, playing a crucial role in maintaining the dNTP pool balance required for DNA synthesis and repair ^[ 4] ^.

GEM molecule has a good stability *in vitro,* and its degradation rate is less than 5% in 24 h at room temperature, but it is rapidly and extensively deaminated by cytidine deaminase (CDA) to the inactive form (2′,2′-difluoro-2′-deoxyuridine, dFdU) in the liver, and its half-life *in vivo* is very short, only 8 to 17 minutes. Therefore, to increase the therapeutic concentration of GEM, a high delivered dose is usually employed; however, this method not only enhances the killing effect of the drug on tumor cells but also increases the toxic effect on normal cells in the body ^[ 5] ^. The concept of prodrug was introduced by Professor Adrien Albert in 1958 ^[ 6] ^, and a prodrug is a biologically inactive derivative of the parent drug molecule, and it exhibits better delivery properties than the parent drug and requires an enzymatic or chemical transformation *in vivo* to release the active drug entity ^[ 7] ^. Prodrug strategies are mainly applied to compounds with poor physicochemical or pharmacokinetic properties. Some prodrugs improve the physicochemical properties ( *e.g.*, water/lipid solubility, chemical stability, *etc.*), and/or pharmacokinetic properties ( *e.g.*, permeability, half-life, *etc.*), while others mainly enhance the targeting properties of the drugs ^[ 8– 9] ^. The main characteristics of targeting prodrugs are precise delivery to the target tissues, and thus the active drugs can be quantitatively transformed and be selective to stay in the target tissues to exert therapeutic effects. Usually, such prodrugs are achieved by targeting unique features of tissues or cells, specific transporters, specific enzymes, antibodies, and gene-encoding enzymes ^[ 10] ^.

Previous studies have shown that tumor cells and platinum-treated tumor cells have high levels of reactive oxygen species (ROS) ^[ 11– 12] ^, and the synthesis of ROS-responsive prodrugs can enhance the release of drugs into tumor cells. H _2_O _2_ is a major component of ROS ^[ 13] ^; therefore, this feature can be utilized to optimize GEM. It has been shown that the α-ketoamide moiety can be broken by H _2_O _2_, and Tang's group ^[ 14]^ designed and synthesized a novel fluorescent 'off-on' probe, Mito-NIRHP, by introducing the α-ketoamide structure into the hemp flower fluorescent group, which can detect the release of H _2_O _2_ with a high sensitivity. However, the use of Mito-NIRHP in the study of H _2_O _2_-activated antitumor prodrugs has been rarely reported.

In the present study, we used the drug combination principle to design and synthesize the prodrug GEM-ZZQ by introducing an H _2_O _2_-sensitive thiazolidinone moiety to the amino group at position 4 of GEM, and examined GEM-ZZQ for chemical structure, stability, and responsiveness to H _2_O _2_. Furthermore, we examined the antitumor effects of GEM-ZZQ in a variety of NSCLC cell lines as well as its effects on normal human epithelial cells, to clarify its advantages in enhancing the targeted anticancer activity in tumor cells and reducing toxicity in normal cells.

## Materials and methods

### Reagents

The H _2_O _2_ solution (30%) was purchased from Jiangsu Yong Hua Chemical Technology Co., Ltd. (Changshu, Jiangsu, China), GEM from Saen Chemical Technology Co., Ltd. (Shanghai, China), trichloroacetic acid from Aladdin (Cat. #T104257, Shanghai, China), Tris-base from Biosharp (Cat. #BS083, Hefei, Anhui, China), and chromatographic methanol from Anhui Shi Lian Special Solvent Co., Ltd. (Anqing, Anhui, China). Ultrapure water was prepared by the ultrapure water system, other reagents were of analytical purity.

### Cell culture

The normal human kidney epithelial cell line, human kidney-2 (HK2), normal human bronchial epithelial cell line (HBE), and human NSCLC cell lines H157, H1299, and H460 were purchased from American Type Culture Collection (ATCC, Rockville, MD, USA), and pancreatic cancer cell line PC-2 was purchased from KeyGEN (Nanjing, Jiangsu, China). All cells were cultured in RPMI-1640 (Cat. #31800-022, Gibco, NY, NYC, USA) medium containing 10% fetal bovine serum (Cat. #FS301-02, TransSerum, Beijing, China). The culture temperature was 37 ℃, and the CO _2_ concentration was 5%.

### Synthesis of GEM-ZZQ

GEM-ZZQ was synthesized through a four-step reaction: a) At 0 ℃, triethylamine was added to the tetrahydrofuran solution of 1,3-thiazolidine-2-one. Then, we slowly added tetrahydrofuran solution of triphosgene to react for 3 h, and collected the organic phase to obtain product 1; b) Gemcitabine hydrochloride, imidazole, 4-dimethylaminopyridine, tert-butyldimethylchlorosilane, and DMF reacted overnight at room temperature to obtain white solid product 2; c) At 0 ℃, pyridine was dropped into the tetrahydrofuran solution of product 2. Then, product 1 was slowly dropped into the above reaction solution. After the reaction at room temperature overnight, the white solid product 3 was obtained; and d) We dropped the tetrahydrofuran solution of tetra-n-butylammonium fluoride into the tetrahydrofuran reaction solution of product 3, and they reacted at room temperature for 3 h to obtain the target compound.

### High-performance liquid chromatography analysis

The high-performance liquid chromatography (HPLC) system was used to separate the samples. HPLC conditions: the chromatographic column was C18 column (46 mm × 250 mm, 5 μm) from Agilent Technologies Inc (Santa Clara, California, USA); mobile phase: water and methanol; flow rate: 1.0 mL/minute; injection volume: 10 μL; elution mode: gradient elution procedure is shown in ***Table 1***.

**Table 1:** Gradient elution procedure

Time (min)	Methanol (%)	Water (%)
0–5	30	70
5–10	50	50
10–20	70	30
20–30	80	20
30–40	90	10

### Analysis of intracellular ROS by flow cytometry

ROS levels were determined by the ROS assay kit (Cat. #S0033S, Beyotime Biotechnology, Shanghai, China). Briefly, cells were incubated with DCFH-DA (10 μmol/L) diluted in serum-free medium for 20 min. The unbounded DCFH-DA was washed out with PBS. Then, cells were trypsinized and resuspended in cold PBS, followed by flow cytometry analysis.

### Construction of osimertinib-resistant NSCLC cell lines

Epidermal growth factor receptor (EGFR) mutant NSCLC cell lines, H1975 and HCC827 were generously provided by Dr. Shi-Yong Sun (Emory University, Atlanta, Georgia, USA). By exposing H1975 and HCC827 cells to gradually increasing concentrations of osimertinib (1 to 1000 nmol/L) for approximately six months, our laboratory newly established H1975 and HCC827 osimertinib-resistant cell lines (H1975-OR and HCC827-OR). Specifically, cells were first exposed to 1 nmol/L osimertinib and then recovered by retreating the drug, when the survival rate dropped to 30%. The dose was initially increased to 5 nmol/L and then to 20 nmol/L and 50 nmol/L, when the IC _50_ was increased 50-fold and 500-fold, respectively. The untreated cells in parallel culture were defined as parental cells (H1975-P and HCC827-P). Cells were cultured in the RPMI-1640 medium containing 10% fetal bovine serum. The culture temperature was 37 ℃, and the CO _2_ concentration was 5% ^[ 15] ^.

### Sulfonyl rhodamine B assay

Cells were seeded in 96-well plates with 2500 cells per well; after 24 h, the cells were treated with different concentrations of GEM/GEM-ZZQ, or the combination of cisplatin and GEM/GEM-ZZQ. After three days, cells were fixed with 10% trichloroacetic acid and stained with sulfonyl rhodamine B (SRB; Cat. #230162, Sigma, St. Louis, MO, USA). Finally, the dye was dissolved in 10 mmol/L Tris buffer, and the absorbance value was measured at 500 nm ^[ 16– 17] ^. IC _50_ and drug combination index (CI) were determined by using Compusyn software ( https://www.combosyn.com) ^[ 18– 19] ^. CI was used to determine the interaction between the drugs. When 0.9 ≤ CI ≤ 1.1, it means that the two drugs are superimposed; when 0.8 ≤ CI < 0.9, it suggests that the two drugs are low synergistic; when 0.6 ≤ CI < 0.8, it indicates a moderate synergistic effect; when 0.4 ≤ CI < 0.6, it represents a high synergistic effect, and when 0.2 ≤ CI < 0.4, it denotes a strong synergistic effect.

### *In vitro* chemical stability assay

Tris-HCl buffer containing 1 mmol/L of GEM-ZZQ at different pH (pH 5, pH 7.4, and pH 9.5) was prepared by ultrasonication. Then, 10 μL of GEM-ZZQ solutions were incubated at 37 ℃ for 2, 4, 6, 8, and 24 h, respectively. And the samples were analyzed by the HPLC method, and the ratio of GEM-ZZQ peak areas was calculated to evaluate the *in vitro* chemical stability of GEM-ZZQ at different times and pH conditions.

### *In vitro* degradation assay

Tris-HCl buffer, containing 1 mmol/L GEM-ZZQ, 40 mmol/L H _2_O _2_, and pH 7.4 was prepared, and 10 μL aliquot solutions were incubated at 37 ℃ for 2, 4, 6, 8, and 24 h, respectively. Thereafter, the samples were tested by HPLC, and the ratio of GEM-ZZQ peak areas was measured to assess the *in vitro* degradation percentage of GEM-ZZQ at different times in 20 mmol/L H _2_O _2_. Similarly, the experimental protocols for the effects of different H _2_O _2_ concentrations ( *i.e.*, 40, 100, 200, and 400 mmol/L) on the *in vitro* degradation of GEM-ZZQ were the same as mentioned above.

### Statistical analysis

Data were shown as the mean ± standard deviation and analyzed with GraphPad Prism 8.0. Student's *t*-test and two-way analysis of variance (ANOVA), followed by the Sidak post-hoc test, were used to determine the significance of the differences. *P* < 0.05 was considered statistically significant.

## Results

### Design and synthesis of GEM-ZZQ

**Figure 1:**
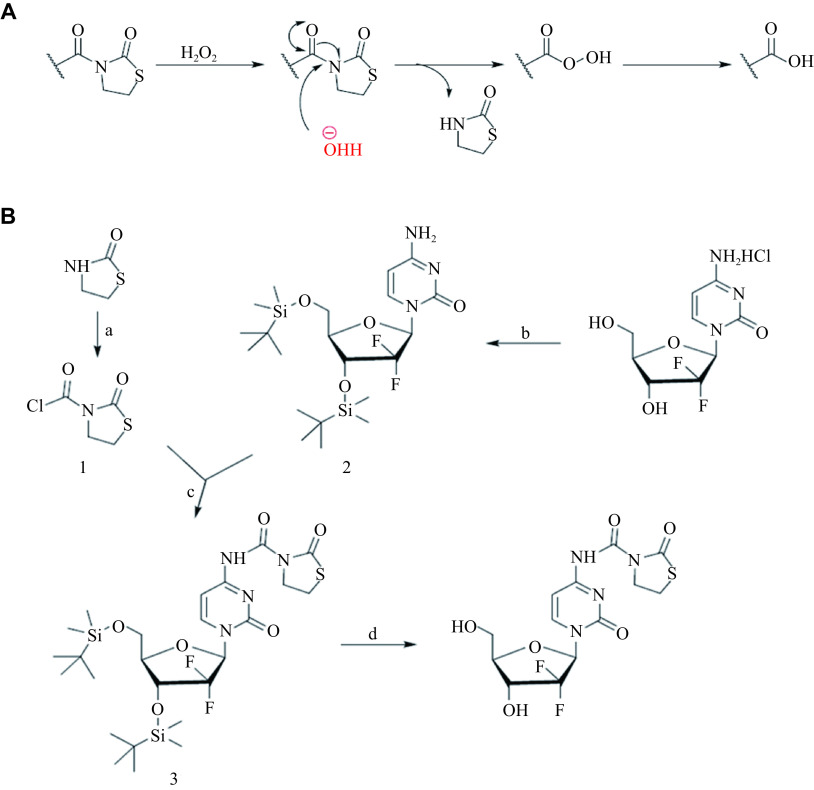
Synthesis of GEM-ZZQ.

The amino group in thiazolidinone moiety can form an amide bond with the carboxyl group and is commonly used to modify drugs containing carboxyl groups. Under the condition of a high concentration of H _2_O _2_, this amide bond can be hydrolyzed to produce free carboxylic acid and therefore form biologically active compounds ( ***Fig. 1A***). We designed the target compound GEM-ZZQ by introducing a thiazolidinone group at the N4 amino group of GEM using the principle of drug collocation design ( ***Fig. 1B***). Tested by ESI-MS, the structure was correct ( ***Supplementary Figs. 1*** and ***2 ***, available online).

### GEM-ZZQ showed a good chemical stability and could respond to H _2_O _2_ *in vitro* to release gemcitabine

**Figure 2:**
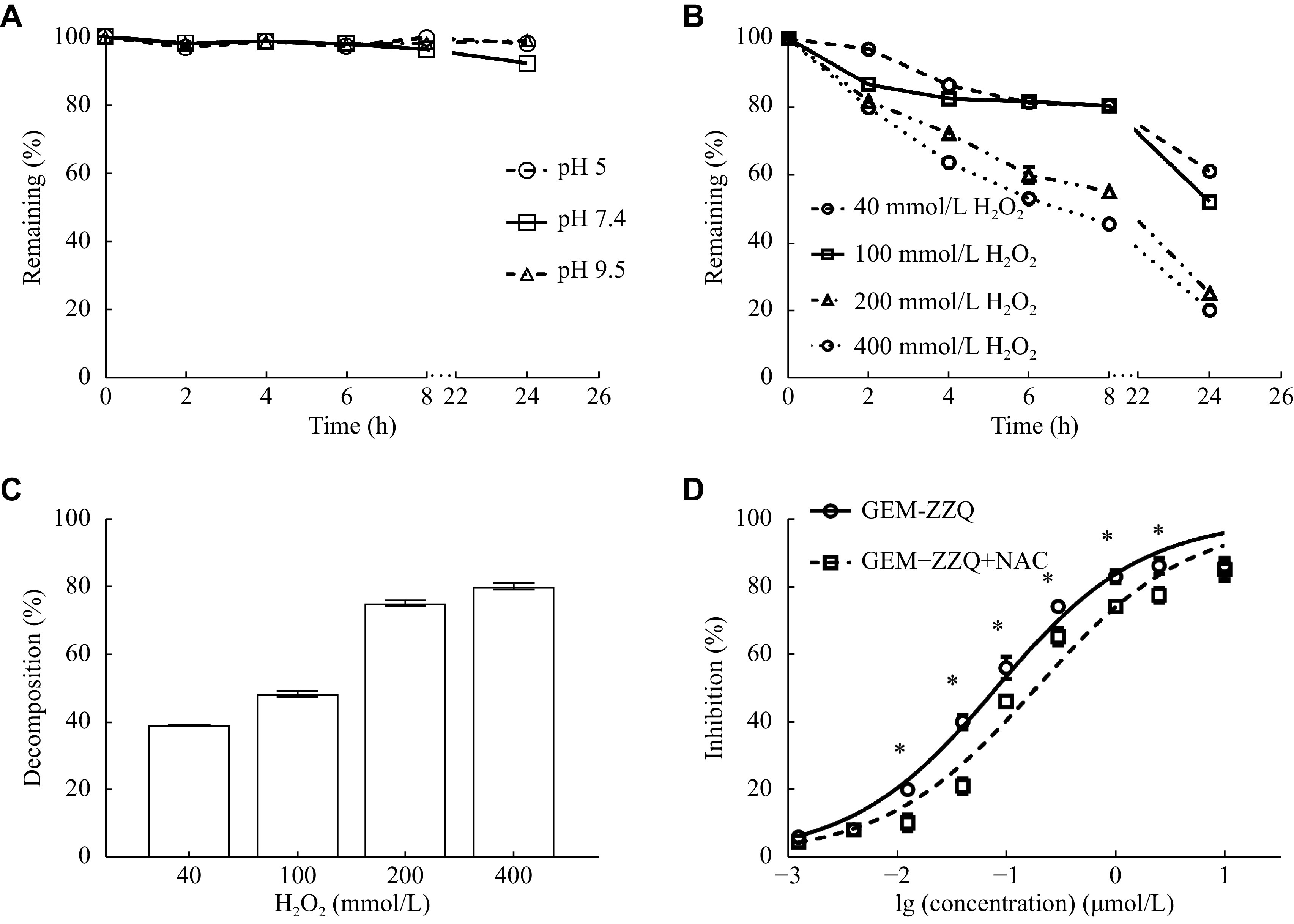
GEM-ZZQ showed good chemical stability and could respond to H _2_O _2_ *in vitro* to release gemcitabine.

Since chemical stability is a key factor affecting the release of prodrugs, we first investigated the *in vitro* chemical stability of GEM-ZZQ. It was found that the residual percentage of GEM-ZZQ could be maintained above 90% after a 24-h incubation in different pH buffer solutions ( ***Fig. 2A***), indicating that GEM-ZZQ has a good *in vitro* chemical stability.

According to our drug design principles, after entering tumor cells, GEM-ZZQ should be broken by a high level of intracellular H _2_O _2_ to release GEM to exert anti-tumor activity and achieve the purpose of tumor targeting. To explore the sensitivity of GEM-ZZQ to H _2_O _2_, the *in vitro* degradation assay of GEM-ZZQ (1 mmol/L) was performed. Our results demonstrated that the degradation rate of GEM-ZZQ reached more than 75% after 24 h in high concentrations of H _2_O _2_ (200 and 400 mmol/L), indicating that GEM-ZZQ was able to respond to H _2_O _2_ and thus released GEM *in vitro* ( ***Fig. 2B*** and ***2C***). Moreover, we pretreated pancreatic cancer cell line PC-2 cells with N-acetylated cysteine (NAC) to scavenge ROS, and our data showed that NAC significantly reduced the inhibitory effect of GEM-ZZQ (IC _50_ = 0.175 [± 0.001] μmol/L) on PC-2 cells, compared with the normal state (IC _50_ = 0.089 [± 0.000] μmol/L) ( ***Fig. 2D***), further indicating that GEM-ZZQ exerts anti-tumor effects *via* H _2_O _2_ activation in tumor cells.

### GEM-ZZQ inhibited the growth of normal human epithelial cells, but the effect was weaker than that of gemcitabine

GEM-ZZQ has the characteristic of releasing GEM in response to intracellular H _2_O _2_. Since the level of intracellular H _2_O _2_ is usually lower in normal tissues than in tumor cells, the GEM level released from GEM-ZZQ should be less in normal cells than in tumor cells, reducing the cytotoxicity in normal cells.

**Figure 3:**
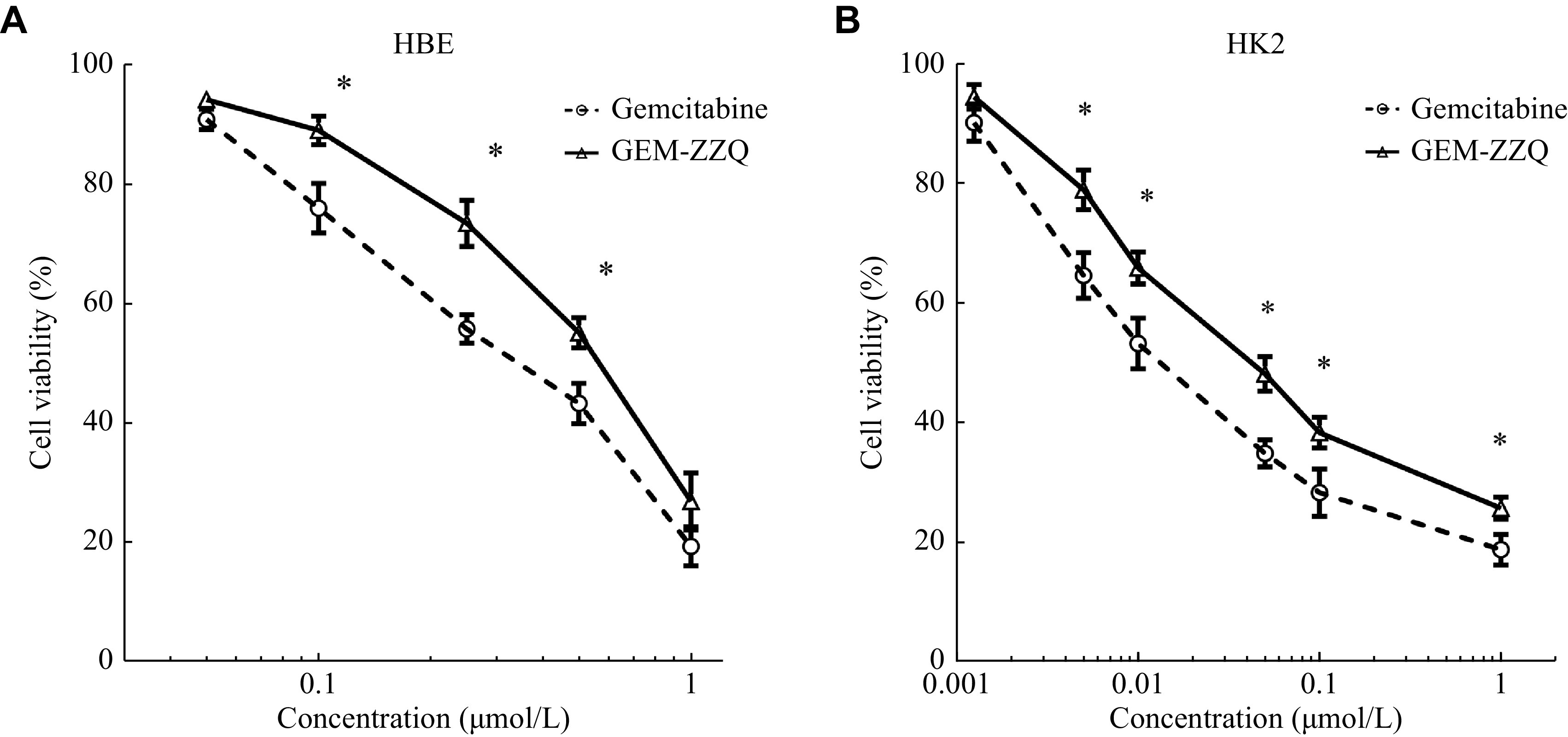
Effect of GEM-ZZQ on normal cell proliferation.

Therefore, we investigated the effect of GME-ZZQ on the growth of HBE and HK2 cells. The SRB assay results showed that GEM-ZZQ and GEM inhibited the growth of HBE cells in a concentration-dependent manner, with IC _50_s of 0.563 (± 0.097) μmol/L and 0.327 (± 0.043) μmol/L, respectively ( ***Fig. 3A***). Similarly, the two drugs also inhibited the growth of HK2 cells, with IC _50_s of 0.041 (± 0.002) μmol/L and 0.016 (± 0.000) μmol/L, respectively ( ***Fig. 3B***). The differences between the inhibition effect of two drugs on HBE and HK2 cells were both statistically significant ( *P* < 0.05; ***Fig. 3A*** and ***3B***). These results indicated that the toxic effects of GEM-ZZQ on normal human bronchial and kidney epithelial cells were lower than those of GEM. In other words, the toxic effects of GEM on normal cells were attenuated to some extent by the thiazolidinedione modification.

### GEM-ZZQ inhibited the growth of several lung cancer cell lines with similar intensity of action to that of gemcitabine

According to the 2022 NCCN NSCLC guidelines, GEM is considered a first-line drug for the treatment of NSCLC. Because the level of ROS is higher in tumor cells than in normal cells, the drugs targeting tumor ROS have a promising development prospect ^[ 20] ^. Therefore, we continued to investigate the inhibitory effect of GEM-ZZQ on NSCLC cell lines.

**Figure 4:**
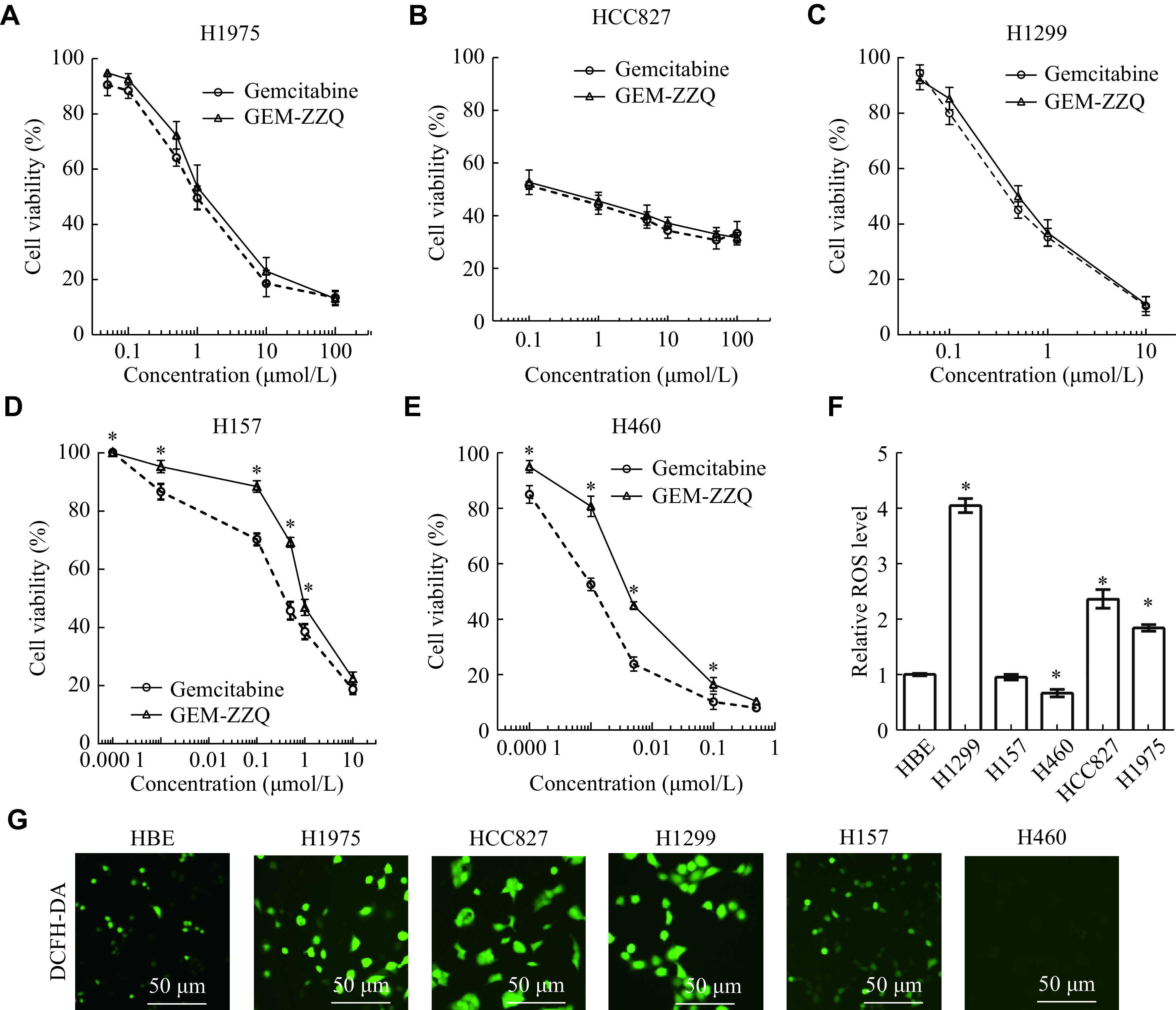
Effect of GEM-ZZQ on the proliferation of NSCLC cell lines.

It was found that GEM-ZZQ had a significant inhibitory effect on the growth of NSCLC cell lines H1975, HCC827, H1299, H157, and H460 ( ***Table 2***), and the inhibitory effect of GEM-ZZQ on tumor growth was in a concentration-dependent manner. It can be also seen that the IC _50_ values of GEM-ZZQ and GEM were similar on NSCLC H1975, HCC827, and H1299 cell lines. Although the IC _50_ values for the H1299 and HCC827 cell lines were statistically significant ( *P* < 0.05), the fold change of the mean IC _50_ was minor, indicating that the two drugs had similar antitumor effects on these NSCLC cells ( ***Fig. 4A***– ***4C***). Meanwhile, we found that the inhibitory effect of GEM-ZZQ on H157 and H460 cells was not as strong as that of GEM, with IC _50_s of 1.061 (± 0.087) μmol/L and 0.451 (± 0.055) μmol/L in H157 cells ( *P* < 0.05), respectively, and 0.004 (± 0.000) μmol/L and 0.001 (± 0.000) μmol/L in H460 cells ( *P* < 0.05), respectively ( ***Fig. 4D*** and ***4E***). The results suggested that GEM-ZZQ could release GEM to inhibit cell growth in multiple lung cancer cells, and its inhibitory effect was comparable to or weaker than that of GEM.

**Table 2:** IC _50_s of gemcitabine and GEM-ZZQ for different lung cancer cell lines

Cell lines	GEM IC _50_ (μmol/L) ^a^	GEM-ZZQ IC _50_ (μmol/L) ^a^	Fold change ^b^
H1975	0.010±0.001	0.014±0.004	1.400
HCC827 ^*^	1. 544±0.069	2.136±0.120	1.383
H1299 ^*^	0.470±0.047	0.567±0.014	1.206
H157 ^*^	0.451±0.055	1.061±0.087	2.353
H460 ^*^	0.001±0.000	0.004±0.000	3.909
Student's *t*-test was used to test the differences between the two groups. ^*^ *P* < 0.05. ^a^Data are presented as mean ± standard deviation, *n* = 3. ^b^Fold change = IC _50_ (GEM-ZZQ)/IC _50_ GEM. GEM indicates gemcitabine. GEM-ZZQ indicates the prodrug with an H _2_O _2_-sensitive thiazolidinone moiety at the 4th amino group of GEM.			

We further analyzed the ROS levels in these cell lines by flow cytometry and fluorescence microscopy. Our results showed that in the cell lines with higher ROS levels (H1299, HCC827, and H1975), GEM-ZZQ exhibited inhibitory effects comparable to that of GEM, while in cells with lower ROS levels (H157 and H460), GEM-ZZQ showed weaker inhibitory effects than that of GEM ( ***Fig. 4F***). These results were also confirmed by fluorescence micrographs ( ***Fig. 4G***). These data indicated that the efficacy of GEM-ZZQ might be correlated with the basal cytoplasmic H _2_O _2_ level and the release of GEM in the cells.

### GEM-ZZQ inhibited the growth of osimertinib-resistant cells, but the effect was weaker than that of gemcitabine

Among the various NSCLC cell lines mentioned above, we noted that HCC827 cells and H1975 cells carried activating mutations of EGFR, such as exon 19 deletion and the point mutations L858R and T790M. EGFR mutations are the second most common oncogenic driver events in NSCLC, and at least 50% of Asian NSCLC patients carry an EGFR activating mutation ^[ 21– 22] ^. The subsequent development of targeted therapy with EGFR tyrosine kinase inhibitors (TKIs), such as a the first-generation (erlotinib, gefitinib), the second-generation (afatinib), and the third-generation (osimertinib) TKIs, has dramatically revolutionized the treatment landscape of NSCLC ^[ 23] ^. However, NSCLC patients inevitably develop the secondary resistance to the treatment within six to 12 months. According to the 2022 NCCN NSCLC treatment guidelines, the first-line therapeutic agent for NSCLC patients carrying the EGFR mutant is usually an EGFR-TKI ^[ 24] ^, while chemotherapy remains one of the first-line pharmacological options available to date for patients with the EGFR-TKI resistance. So, we next investigated the growth inhibitory effects of GEM-ZZQ on H1975-OR and HCC827-OR of NSCLC cells that were resistant to osimertinib.

**Figure 5:**
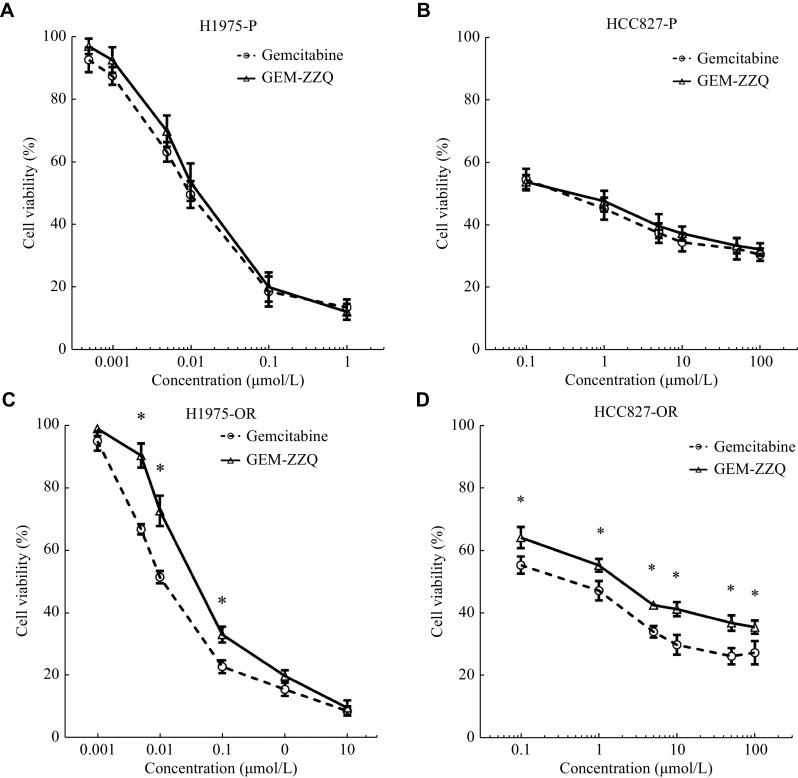
Effect of GEM-ZZQ on the proliferation of NSCLC with EGFR-TKI resistance cell lines.

There is no difference between the growth inhibitory effect of GEM-ZZQ and GEM on both H1975 and HCC827 NSCLC parent cell lines ( *P* > 0.05) ( ***Fig. 5A*** and ***5B***). Interestingly, our results showed that the inhibitory effect of GEM-ZZQ on H1975-OR was inferior to that of GEM, with IC _50_s of 0.043 (± 0.003) μmol/L and 0.012 (± 0.001) μmol/L, respectively ( *P* < 0.05) ( ***Fig. 5C***); the growth inhibitory effect of GEM-ZZQ on HCC827-OR cells was also weaker than that of GEM, with IC _50_s of 4.306 (± 0.065) μmol/L and 1.338 (± 0.020) μmol/L ( ***Fig. 5D***), respectively ( *P <*0.05). Our data suggested that GEM-ZZQ could inhibit the growth of osimertinib-resistant NSCLC cells, but not as effective as GEM. Previous studies showed that the ROS levels in drug-resistant cells were lower than in sensitive cells ^[ 25] ^, which may be the reason why GEM-ZZQ showed a weaker inhibitory effect on the growth of drug-resistant NSCLC cells than that of GEM.

### GEM-ZZQ in combination with cisplatin showed a better synergistic effect than gemcitabine

Data from preclinical studies suggested a synergistic effect between GEM and platinum compounds, such as cisplatin or carboplatin ^[ 26– 27] ^. Considering that cisplatin can significantly increase intracellular H _2_O _2_ levels and that the cleavage and activation of GEM-ZZQ require a high level of H _2_O _2_, we investigated whether GEM-ZZQ had a better synergistic effect with cisplatin.

**Figure 6:**
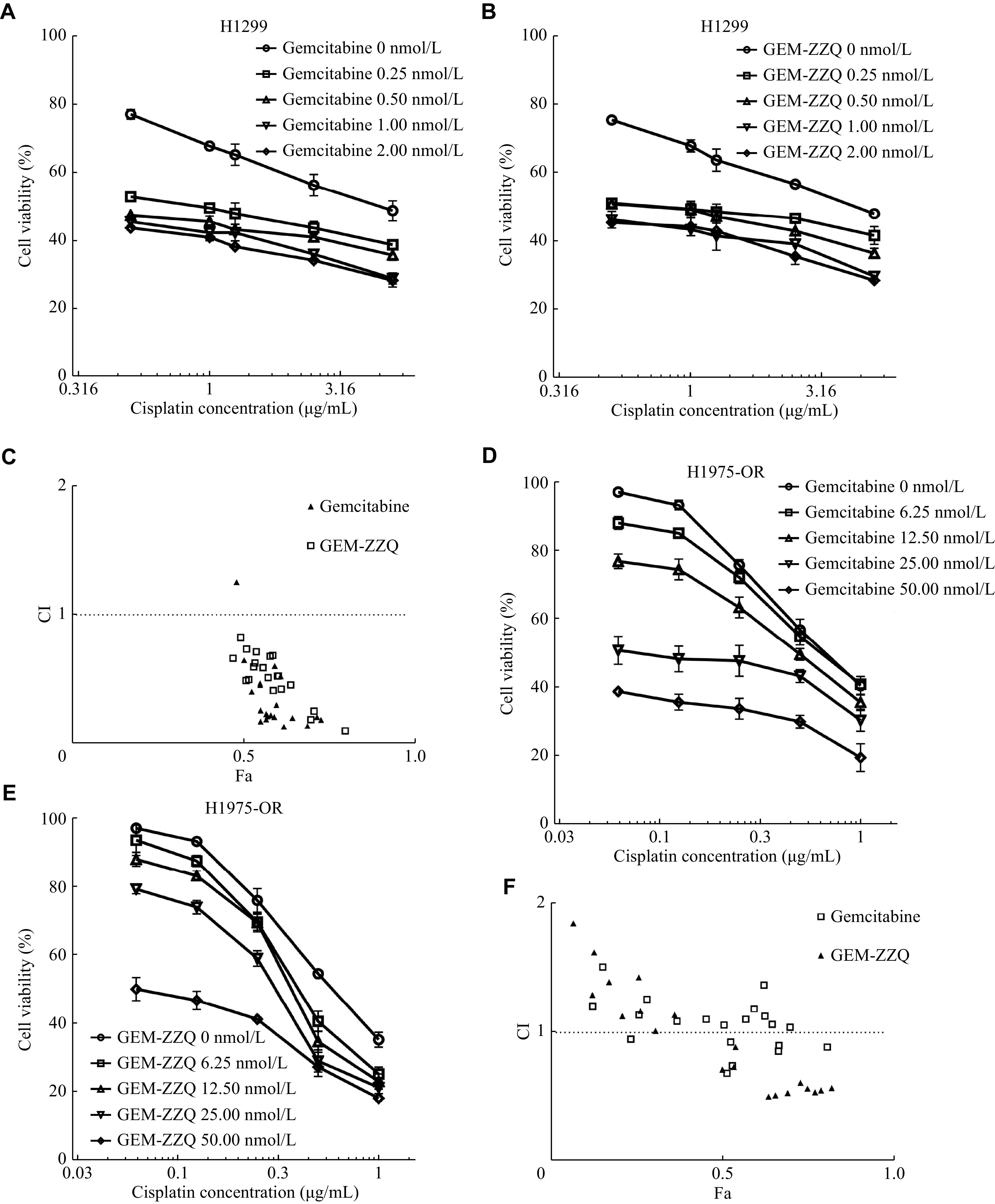
GEM-ZZQ showed a synergistic effect with cisplatin in NSCLC with EGFR-activating mutations.

The results of the SRB assay showed that both GEM and GEM-ZZQ in combination with cisplatin were synergistic on H1299 cells ( ***Fig. 6A***– ***6C***). We also observed similar IC _50_ for cisplatin in combination with GEM and GEM-ZZQ, with no significant differences ( *P* > 0.05) ( ***Table 3***).

**Table 3:** IC _50_s of cisplatin when it was administrated in combination with GEM and GEM-ZZQ in H1299 cell lines

GEM/ GEM-ZZQ (μmol/L)	Combined GEM (μg/mL) ^a^	Combined GEM-ZZQ (μg/mL) ^a^	Fold change
0	2.895±0.055	2.825±0.057	0.976
0.25	1.204±0.048	1.272±0.080	1.056
0.50	0.964±0.037	1.142±0.118	1.185
1.00	0.809±0.032	0.855±0.086	1.057
2.00	0.721±0.024	0.835±0.071	1.158
Student's *t*-test was used to test the differences between the two groups. ^a^Data are presented as mean ± standard deviation, *n* = 3. Fold change = IC _50_ (GEM-ZZQ)/IC _50_ (GEM). GEM-ZZQ indicates the prodrug with an H _2_O _2_-sensitive thiazolidinone moiety at the 4th amino group of GEM. Abrreviation: GEM, gemcitabine.			

According to the 2022 NCCN guidelines for the management of NSCLC patients, the combination of platinum compounds with GEM is the first-line treatment option for osimertinib-resistant patients. Therefore, we further investigated the effect of the drug combination in the above-mentioned osimertinib-resistant cell lines H1975-OR. The results showed that the combination of cisplatin with GEM was not synergistic (CI > 1) or only additive (0.9 ≤ CI ≤ 1.1) at most doses; meanwhile, the combination of cisplatin with GEM-ZZQ showed a better synergistic effect ( ***Fig. 6D***– ***6F***). Moreover, the IC _50_ of cisplatin was lower in combination with GEM-ZZQ in two low-dose groups (0.006 μmol/L and 0.013 μmol/L), suggesting a better effect ( ***Table 4***). One possible explanation for our findings is that osimertinib-resistant cells did not respond well to GEM-ZZQ due to the low intracellular H _2_O _2_ levels, but when in combination with cisplatin, the level of intracellular H _2_O _2_ was increased and then GEM-ZZQ showed better synergistic effects than GEM.

**Table 4:** IC _50_s of cisplatin when it was administrated in combination with GEM and GEM-ZZQ in H1975-OR cell lines

GEM/ GEM-ZZQ (nmol/L)	Combined GEM (μg/mL) ^a^	Combined GEM-ZZQ (μg/mL) ^a^	Fold change
0	0.730±0.055	0.741±0.049	1.015
6.25 ^*^	0.642±0.020	0.405±0.029	**0.631**
12.50 ^*^	0.437±0.029	0.369±0.018	**0.844**
25.00 ^*^	0.175±0.017	0.287±0.011	1.640
50.00 ^*^	0.076±0.007	0.119±0.009	1.566
Student's *t*-test was used to test the differences between the two groups. ^*^ *P* < 0.05. ^a^Data are presented as mean ± standard deviation, *n* = 3. Fold change = IC _50_ (GEM-ZZQ)/IC _50_ (GEM). Bold text indicates a better synergistic effect compare to the group Comb GEM. GEM-ZZQ indicates the prodrug with an H _2_O _2_-sensitive thiazolidinone moiety at the 4th amino group of GEM. Abbreviation: GEM, gemcitabine; OR, osimertinib-resistant.			

## Discussion

GEM is a pyrimidine nucleoside analog that belongs to the antimetabolic class of antitumor drugs with a broad-spectrum antitumor activity, and is currently used in the treatment of lung and pancreatic cancer ^[ 28] ^. GEM can be rapidly deaminated by CDA with a short plasma half-life, and is often administered clinically at high doses (as high as 1000 mg/m²). With pharmacokinetic characteristics of a low specificity and selectivity and a short half-life, GEM shows non-specific effects *in vivo*, causing toxic reactions ^[ 5] ^.

Previously, many studies focused on modifying GEM molecules to improve the targeting of tumor cells and reduce toxicity to other tissues and organs. In recent years, it was found that chemical modification of two important positions in the GEM molecule, the fourth amino group and the fifth hydroxyl group, could protect against the deamination of CDA and improve molecular stability as well as prolong the half-life ^[ 29] ^. In the present study, the target compound GEM-ZZQ was designed and synthesized by introducing an H _2_O _2_-sensitive thiazolidinone moiety to the fourth amino group of GEM, and the results of preliminary pharmaceutical chemistry experiments confirmed that GEM-ZZQ had a good *in vitro* chemical stability and could release GEM in response to a high level of intracellular H _2_O _2_.

Considering that tumor cells usually have higher levels of ROS, such as H _2_O _2_, peroxides, and superoxides, compared with normal cells, the prodrug GEM-ZZQ can release more GEM in response to H _2_O _2_ in tumor cells and exert stronger cytotoxic effects, while in normal cells, a small amount of GEM is released, thus reducing the toxic response. Our results showed that in normal epithelial cells (HK2 and HBE), the inhibitory effect of GEM-ZZQ on cell growth was significantly lower than that of GEM, indicating that the toxic effect of the prodrug GEM-ZZQ modified by thiazolidinedione on normal cells was reduced. Our study on several strains of NSCLC cells revealed that the growth inhibitory effect of GEM-ZZQ was similar to that of GEM in H1299, H1975, and HCC827 cells. However, on H157 and H460 cells, GEM-ZZQ was not as effective as GEM, which may be correlated with the lower level of H _2_O _2_ in these cells. These results indicated that the prodrug GEM-ZZQ could be hydrolyzed to the active form ( *i.e.*, GEM), but it is not effective in all lung cancer cells, and its action strength may be correlated with cell intracellular H _2_O _2_ levels.

Clinically, GEM remains one of the first-line therapeutic agents for cancer patients who have developed resistance to EGFR-TKI. Studies have reported a change in the metabolic profile of resistant cells, compared with sensitive cells, with an increase in reduced glutathione levels, and a decrease in the levels of ROS and H _2_O _2_ in resistant cell lines ^[ 25] ^. In the present study, we found that the growth inhibition effect of GEM-ZZQ was significantly weaker than that of GEM on the *in vitro* cultured osimertinib-resistant cell line H1975 (H1975-OR), which indirectly suggested that the effect of GEM-ZZQ was correlated with the level of intracellular H _2_O _2_.

In summary, we designed and synthesized a thiazolidinone-modified GEM, GEM-ZZQ, which can release GEM in response to the high level of intracellular H _2_O _2_ in tumor cells. The prodrug GEM-ZZQ had a better *in vitro* chemical stability and H _2_O _2_ responsiveness. Compared with GEM, GEM-ZZQ also had less toxic effects on normal human epithelial cells and a similar growth inhibition effect on various NSCLC cell lines as well as superior synergistic effects with cisplatin in the low dose on EGFR-TKI osimertinib-resistant cell lines. Our study may provide some clues for developing new effective drugs for the treatment of lung cancer and new ideas for future design and development of prodrugs responding to H _2_O _2_, shedding some light on the prodrug rational design.
